# Measuring health-related quality of life in patients with conservatively managed stage 5 chronic kidney disease: limitations of the Medical Outcomes Study Short Form 36: SF-36

**DOI:** 10.1007/s11136-016-1313-7

**Published:** 2016-08-13

**Authors:** Gilli Erez, Lucy Selman, Fliss E. M. Murtagh

**Affiliations:** 1 Palliative Care Team, Northwick Park Hospital, London North West Healthcare NHS Trust, Watford Road, Harrow, London, HA1 3UJ UK; 2 Department of Palliative Care, Policy and Rehabilitation, Cicely Saunders Institute, King’s College London, London, UK

**Keywords:** Chronic kidney diseases, Quality of life, Short Form 36 Health Survey, Longitudinal studies

## Abstract

**Purpose:**

Chronic kidney disease (CKD) negatively affects health-related quality of life (HRQoL), which is often measured using the Medical Outcomes Study Short Form 36 (SF-36) questionnaire. However, the adequacy of SF-36 in this population has not been reported. We aimed to determine floor and ceiling effects and responsiveness to change of SF-36 in patients with conservatively managed stage 5 CKD.

**Methods:**

SF-36 data were collected prospectively. Floor and ceiling effects were estimated for each SF-36 scale and summary measure based on raw scores. The minimal clinically important difference (MCID) was estimated using a combination of anchor-based and distribution-based methods. Responsiveness to change was assessed by comparing MCID for each scale and summary measure to its smallest detectable change.

**Results:**

SF-36 data were available for 73 of the 74 study participants. Using baseline data, floor and/or ceiling effects were detected for 3 of the 8 SF-36 scales. The anchor-based estimation of MCID based on differences in baseline functional status yielded the most reliable results. For the physical component summary, MCID was estimated at 5.7 points. Whilst the two SF-36 summary measures were responsive to change and free of floor and/or ceiling effects, six of the eight scales were not.

**Conclusions:**

This small study of patients with conservatively managed stage 5 CKD found that only the summary measures of SF-36 and 2 of its 8 scales can be used to assess changes in HRQoL over time. These findings suggest that in this population, alternative HRQoL assessment tools should be considered for future studies.

## Background

The health-related quality of life (HRQoL) of patients with chronic kidney disease (CKD) is poorer than that of the general population, both in the early stages of CKD [1–5] and in advanced (stage 5) disease [6–9]. Stage 5 CKD is diagnosed when kidney function, measured by the estimated glomerular filtration rate (eGFR), falls below 15 mL/min/1.73 m^2^ [10]. Its prevalence and incidence in the developed world are increasing [11–13]. In the UK, for example, prevalence has increased from 523 per million population (pmp) in 2000 to 861 pmp in 2012, and incidence rates have increased from 95 pmp in 2001 to 108 pmp in 2012 [11, 12]. In particular, prevalence in the over 85 age group is rising steeply and nearly doubled between 2006 and 2012 [11].

Stage 5 CKD is a life-limiting disease for which renal replacement therapy (RRT) is often recommended. However, RRT imposes a significant burden on those requiring it, with implications for the physical and social lives of patients and their carers [14–16]. The survival advantage of RRT, and specifically that of dialysis treatment, appears to be limited to patients without multi-morbidity [17–23]. Therefore, conservative management of stage 5 CKD is increasingly offered to selected patients [24], with a focus on best supportive care, often with input from palliative care services which actively manage symptoms and provide holistic care [21, 25].

Most studies of HRQoL in patients with stage 5 CKD use cross-sectional methods, and the few longitudinal studies yield conflicting results [6, 7, 15, 26]. Moreover, most studies focus on dialysis patients; therefore, little is known about the HRQoL of conservatively managed patients and its change over time [27, 28]. The interpretation of any report of HRQoL relies, however, on the validity and reliability of the tools used.

The Medical Outcomes Study Short Form 36 (SF-36) is a widely used HRQoL questionnaire which has been extensively validated in CKD populations, and is commonly used either in its original generic form or as part of the kidney disease quality of life (KDQOL) questionnaire developed for dialysis patients [29, 30]. SF-36 has also been used to measure the HRQoL of patients with advancing disease who are approaching death [7, 26], but to our knowledge its suitability in this population has not been tested.

The appropriateness of a HRQoL measurement tool relies in part on its measurement properties, including floor and/or ceiling effects and responsiveness to change, and on its interpretability [31]. Floor and/or ceiling effects are present when >15 % of the population score lowest or highest, respectively, on a certain scale [32]. When present, a tool cannot differentiate between people who may have had significantly different experiences not captured by the research tool. Responsiveness to change refers to the ability of a scale to detect clinically important changes over time [32]. Interpretability, the degree to which qualitative meaning can be assigned to quantitative scores [33] is also vital, because the magnitude and statistical significance of a reported change do not necessarily correlate with clinical relevance. An advantage of SF-36 is that as part of its scoring process, individual scores are compared to a reference (‘normative’) population. This facilitates intuitive interpretation of scores as ‘higher’ or ‘lower’ than average in the general population and enables indirect comparison of normed results between different studies [34]. In many populations, SF-36 is regarded sensitive to change, but robust estimations of its minimal clinically important difference (MCID) and interpretability are lacking.

The aim of the current study is to estimate floor and ceiling effects and responsiveness to change of SF-36 in patients with conservatively managed stage 5 CKD, to reflect on its appropriateness as a HRQoL measurement tool in this growing patient population.

## Methods

This was a secondary data analysis. We used cross-sectional baseline data available from a primary study conducted by one of the authors (FM) [35]. Primary data were collected prospectively and longitudinally from participants recruited from three renal units in London and South-East England between April 2005 and November 2006. All three units had dedicated multi-disciplinary services for conservative management, offering needs-based physical, psychological and social support. Inclusion criteria were a diagnosis of stage 5 CKD with a confirmed decision for conservative management. The only exclusion criterion was lack of capacity to give consent to participate in the study. Potential participants were referred to the researcher (FM) by the clinical teams, following which informed consent was sought. Continued consent was obtained monthly by telephone.

**Fig. 1:**
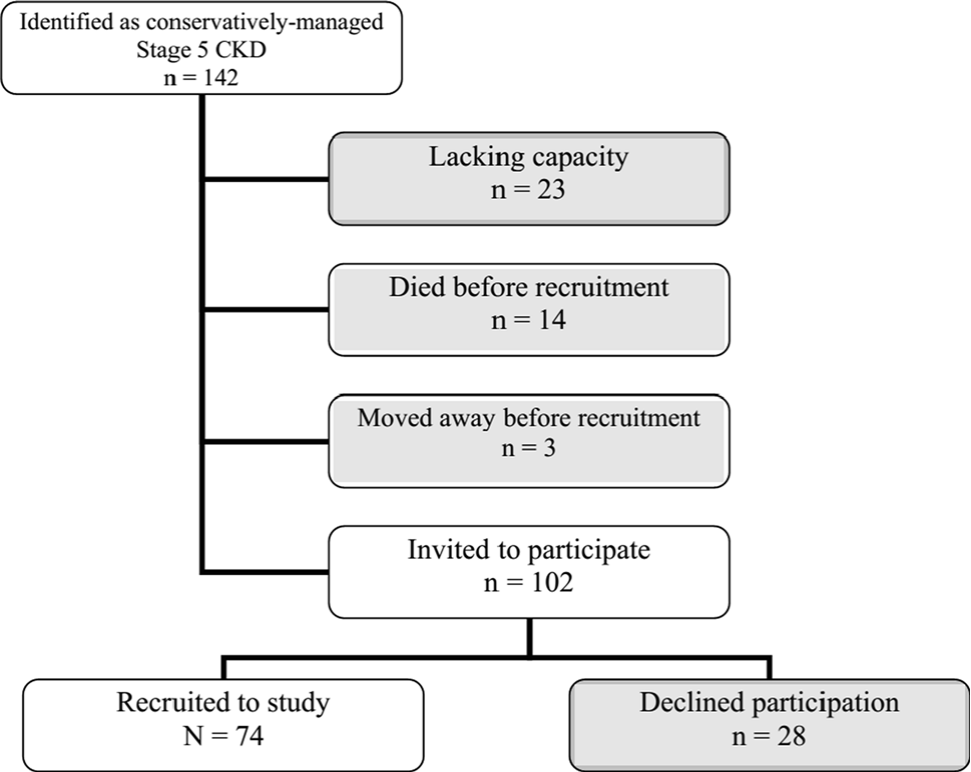
Recruitment flow chart (adapted from the CONSORT flow diagram [36])

One hundred and forty-two people were identified as potential participants. Of those, 40 were excluded, as shown in Fig. 1 [36]. Of the 102 remaining patients, 74 consented to participate and formed the final sample for the study. Clinical characteristics were similar between participants and non-participants, as were age and sex. Ethnicity was distributed unequally with a higher level of participation among those from minority ethnic groups as compared with white patients. Further details of the primary study were previously reported [35, 37, 38].

Baseline clinical and demographic data were collected from clinical files. Data regarding HRQoL were collected at baseline and every 3 months using a postal standard SF-36 questionnaire, which assesses participants’ HRQoL over the preceding 4 weeks. Data collection continued until death, withdrawal from the study or end of study.

The primary study had ethical approval from King’s College Hospital NHS Research Ethics Committee (number 04-03-092). Specific approval for this further analysis was waived by the Ethics Committee as it did not diverge from the original research to which participants consented.

### Scoring participants’ responses to SF-36

SF-36 items were coded and scored as outlined in the SF-36 scoring manual [39]. Briefly, after appropriate recoding of complete and missing data, raw scores of the 8 scales [physical functioning (PF), role physical (RP), bodily pain (BP), general health (GH), vitality (VT), social functioning (SF), role emotional (RE) and mental health (MH)] were computed and transformed to a 0–100 scale, with higher scores representing better HRQoL. Norm-based transformation was carried out for each scale, and the two summary measures [physical component summary (PCS) and mental component summary (MCS)] were computed. Norm-based scoring yields a distribution of scores in which scores lower than 50 indicate poorer HRQoL compared to the reference (‘normative’) population, which in turns enables a more intuitive interpretation of the impact of the condition being examined, compared with the reference population. For this study, data from the Welsh Health Survey 2007 were used as reference [40, 41].

### Selecting a normative population

The study population was an elderly population drawn from London and South-East England. An ideal normative data set would be drawn from the same area at the same time, but include similarly aged patients without CKD or with early-stage CKD. Unfortunately, such a normative data set was not available. The Welsh Health Study (WHS), drawn from the work of Burholt and Nash [40], but originally derived from the National Centre for Social Research on behalf of the Welsh Assembly Government [41], was chosen because data were collected in the UK at a contemporary time period and included elderly participants. HRQoL is known to be affected by age, but normative data for elderly participants are scarce [34]. We aimed to overcome this limitation by using as reference only WHS data which were derived from people of similar age to our sample (mean age 80.7 ± 6.8, median 81.4 and 25th percentile of 78.1). Weighted means and standard deviations (SDs) were calculated for WHS participants aged 75 and over. Those means and SDs were then used to calculate the study normed scores [39]. WHS data were drawn from a random sample of private households in Wales. Although not explicitly reported by Burholt and Nash [40], a potential disadvantage of WHS data was that ethnicity in this sample was likely to be predominantly white [42].

### Handling of missing data

Missing items and missing questionnaires were differentially coded and reported. Handling of specific SF-36 missing items followed the SF-36 scoring manual [39]. The manual suggests that missing items can be estimated by a single imputation method if they contribute to <50 % of a given scale, and the imputation method is provided. If ≥50 % of items comprising a scale are missing, then the scale cannot be calculated and is regarded as missing.

### Study outcomes

The primary outcome for this study was the PCS of the SF-36, which was selected as it has been shown to be most responsive to treatments that change physical morbidity [43]. Secondary outcomes were the MCS and the 8 SF-36 scales. The PF scale, the main contributor to PCS, was chosen as the key secondary outcome, as the developers of the SF-36 tool emphasise that despite the strengths of the component scores, they may not be as valid as a scale, especially when differences are concentrated in one scale [44].

### Estimation of floor and/or ceiling effects

Floor and/or ceiling effects have been defined as present when >15 % of the population score lowest or highest, respectively, on a certain scale [32]. Floor and ceiling effect estimations for each of the 8 SF-36 scales were based on raw scores, as suggested by the manual [39]. For PCS and MCS, the lowest possible score was arbitrarily assumed to be either 10 or 20, as suggested by Taft et al. [45].

### Estimation of responsiveness to change

Responsiveness to change refers to the ability of a scale to detect clinically important changes over time [32]. Its estimation is based on comparing the smallest detectable change (SDC), i.e. the smallest change which exceeds the measurement error of the tool, to the MCID, i.e. the smallest difference in a scale that patients perceive to be beneficial [46]. A tool can only be deemed sensitive to change if its MCID exceeds its SDC [32].

SDC was calculated using the formula: ${\text{SDC}} = 1.96 \times \surd 2 \times {\text{SEM }}$ (where SEM is the standard error of measurement) [32].

MCID can be estimated by a variety of methods, each with its advantages and inherent limitations, as reviewed by Crosby et al. [47]. Of those, we chose three methods which could be applied to our data and planned to compare estimations yielded by them. The method yielding the most stringent criteria was planned to be selected for further analyses. Anchor-based cross-sectional methods use a difference which is known to be clinically significant between two populations at one point in time, in order to estimate the minimal amount of change needed within one population over time which would be clinically significant.

1. The anchor-based cross-sectional method of comparison to disease-related criteria:

The extent to which SF-36 scales and summary measures reflect differences between known contrasting groups was assessed following a method described by Cella et al. [48] Contrasting groups were chosen if they had been shown to influence SF-36 scores in the general English population (age, gender, comorbidities) [34], or if they had previously been shown to correlate with prognosis in this study’s population (performance status, eGFR) [38]. We used previously established assessment methods to quantify these domains: Karnofsky performance scale (KPS) [30, 49] for functional status, and both Charlson [50] and Davies [51] scales for comorbidities. Further justification for the use of those methods is provided elsewhere [35]. Groups were dichotomized across the median for each scale and summary measure and compared using the Mann–Whitney *U* test. Where significant differences were found, means were adjusted to age and gender using an ANCOVA model.

2. The anchor-based cross-sectional method of comparison to a reference population:

We adjusted the method described by Jacobson and Truax, which is based on the assumption that there is a population which can be considered ‘functional’, i.e. normative, whereas the study population at baseline is considered ‘dysfunctional’ [52]. Mean scores for the functional and dysfunctional populations thus serve as anchors. A *c* value is calculated, beyond which the test score would be closer to the normative population mean than to the study mean. The equation is: $c = \left( {S0M1 + S1M0} \right)/\left( {S0 + S1} \right)$, when M0 and S0 are mean and SD, respectively, for the normative (‘functional’) population, and M1 and S1 are mean and SD, respectively, for the study (‘dysfunctional’) population. We assumed that if a score obtained over time in the study population is closer to that of the ‘functional population’ at baseline than it is to the study population at baseline, i.e. it is beyond the *c* value cut-off, then this change is clinically significant. The amount of change can only be calculated in relation to the anchors, but the direction of change is irrelevant to its clinical significance; therefore, we used the formula:

$${\text{MCID }} = \left| {{\text{study}}\,{\text{mean}} {-} c} \right|$$

High-quality SF-36 data from people with less advanced CKD were not available; therefore, WHS 2007 data for people aged 75 and over were used as the normative population [40]. Study SF-36 transformed (not normed) scores were used for these comparisons. *c* value was determined for each SF-36 scale, and MCID was calculated using the equation above.

3. The distribution-based method based on SEM:

SEM, the standard error of measurement, is a sample-independent measure which can be used to reflect a tool’s accuracy, i.e. differentiate between a true difference in scores and one that is due to measurement error [32]. It is also used to calculate the SDC, which in turn can be compared to the MCID to assess the tool’s responsiveness to change. Not all authors consider the SDC, and some have suggested that 1 SEM is an acceptable approximation of the MCID [46, 48]. Based on previous data for SF-36, [39] and on generally acceptable standards [32, 53], reliability (*r*) in the current study was estimated using Cronbach’s alpha for internal consistency reliability. SEM was estimated by the equation: ${\text{SEM}} = SD \times \surd \left( {1 - r} \right)$.

MCID was estimated using baseline SF-36 data and then re-estimated using the last available SF-36 data for each participant.

### Data analysis

IBM-SPSS version 22 was used for data analysis [54]. As all data were not normally distributed, nonparametric descriptive statistics and tests were used throughout the analysis; however, as the tests compare means rather than medians, both means and medians are reported. Differences between groups were regarded as statistically significant if the two-tailed *p* value was smaller than 0.05. The Bonferroni method of adjusting for multiple comparisons was applied when primary comparisons yielded statistically significant results.

## Results

38 men and 36 women were recruited. 51 of the 74 participants (68.9 %) were of white ethnicity. Mean age was 80.7 (±6.8) years, and mean eGFR was 11.67 (±2.75) mL/min/m^2^. Participants were followed up for a mean of 209 (±152.5) days (range 0–630 days) after enrolment. Baseline characteristics of the study population appear in Table 1.

**Table 1:** Baseline characteristics of the study population (*N* = 74)

Study site, *N* (%)	
Site 1	34 (45.9)
Site 2	31 (41.9)
Site 3	9 (12.2)
Age (years)	
Mean (SD)	80.7 (6.8)
Median (IQR)	81.4 (78.1–85.2)
Sex, *N* (%)	
Male	38 (51.4)
Female	36 (48.6)
Ethnicity, *N* (%)	
White	51 (68.9)
Black	12 (16.2)
South Asian	6 (8.1)
Other	5 (6.8)
eGFR	
Mean (SD)	11.67 (2.75)
Median (IQR)	12.25 (9.73–14.18)
Davies comorbidity index, *N* (%)	
Grade 0	14 (18.9)
Grade 1	44 (59.5)
Grade 2	16 (21.6)
Charlson comorbidity index	
Mean (SD)	4.4 (2.06)
Median (IQR)	4.0 (3–5.25)
Karnofsky performance status	
Mean (SD)	62 (11.2)
Median (IQR)	60 (50.0–70.0)

SF-36 data were available for 73 participants (98.6 %). There were no missing SF-36 items in the baseline measurements used for the current analysis. Nine of 2628 (0.34 %) items were missing in the analysis of last available SF-36 data. The characteristics of the 8 SF-36 scales and 2 summary measures at baseline are presented in Table 2. Floor and/or ceiling effects at baseline were detected for 3 of the 8 scales (RP, RE and BP) and were not detected for the summary measures PCS and MCS.

**Table 2:** Characteristics of SF-36 scales and summary measures at baseline (*N* = 73)

SF-36 scale	Normed scores		Raw scores		
	Mean (SD)	Median (IQR)	Possible range	Observed range	Floor/ceiling effects
Physical functioning	44.0 (6.4)	42.5 (39.4–47.2)	10–30	10–26	No
Role physical	44.6 (6.0)	45.3 (39.8–48.9)	4–20	4–17	Floor effect (19 % scored 4)
Bodily pain	53.6 (9.7)	52.4 (45.3–65.3)	2–12	3.2–12	Ceiling effect (30 % scored 12)
General health	41.2 (8.1)	41.4 (34.9–46.2)	5–25	5–21.4	No
Vitality	41.5 (8.7)	39.9 (34.3–48.2)	4–20	4–15	No
Social functioning	46.2 (8.7)	45.1 (41.3–52.7)	2–10	2–10	No
Role emotional	49.8 (8.9)	55.0 (42.8–57.4)	3–15	3–15	Ceiling effect (49 % scored 15)
Mental health	48.7 (9.8)	50.6 (44.0–56.0)	5–25	9–25	No
PCS	44.6 (5.8)	44.1 (40.9–47.8)	N/A	N/A	No
MCS	48.1 (8.8)	49.8 (42.4–54.9)	N/A	N/A	No

*SD* standard deviation, *IQR* inter-quartile range, *PCS* physical component summary (of SF-36), *MCS* mental component summary (of SF-36), *N/A* not applicable

Mean baseline values of each SF-36 scale and summary measure were compared by gender and across median values for age, KPS, Davies comorbidity index, Charlson comorbidity index and eGFR. For all scales and summary measures, and following Bonferroni adjustment for those six comparisons, mean scores were significantly different across performance status groups. MCID (i.e. within-group difference over time) was estimated as the difference between those KPS means (at one point in time), adjusted for age and sex (Table 3). Comparisons based on other criteria yielded insignificant differences between groups (at one point in time) and could therefore not be used to estimate MCID.

**Table 3:** Summary of estimations of MCID using the anchor-based and distribution-based approaches (with baseline data)

	Comparison to disease-related criteria (KPS)	Comparison to normative data^a^	SEM	SDC
PCS	5.7	N/A	1.63	4.52
MCS	9.2	N/A	2.46	6.82
Physical functioning	7.9	7.4	2.24	6.21
Role physical	4.3	7.0	1.77	4.91
Bodily pain	7.1	5.2	2.71	7.51
General Health	8.2	9.2	3.61	10.01
Vitality	8.3	8.9	3.37	9.34
Social functioning	7.9	5.7	3.20	8.87
Role emotional	8.2	0.4	2.22	6.15
Mental health	9.7	1.3	3.42	9.48

*KPS* Karnofsky performance scale, *SEM* standard error of measurement, *SDC* smallest detectable change, *PCS* physical component summary, *MCS* mental component summary, *N/A* not applicable

^a^In this column, scores are transformed (0–100 scale) but not normed

Table 3 compares MCID estimations derived by the different approaches: the anchor-based method based on KPS, the anchor-based method using *c* values (with WHS data as a comparative population) and MCID estimation based on the distribution-based method (SEM and SDC)—all using baseline data. As planned, we chose the method yielding the most stringent criteria for our final estimation of MCID. For PCS, MCID was thus estimated as 5.7 points, and for MCS MCID was estimated as 9.2 points.

SEMs were consistently smaller than anchor-based estimations of MCID. Further, SDCs for five of the eight scales (RP, BP, GH, VT and SF) were higher than MCIDs, suggesting that those scales are not sensitive to change in this study population. The summary scores PCS and MCS, and the scales PF and MH were sensitive to change and free from floor or ceiling effects in this population.

Re-estimation of floor and/or ceiling effect and MCID using last available data for each of the 73 participants was based on data collected a median of 29 days prior to last contact (or median of 40 days prior to death in the 49 participants who died during the study). For all scales and summary measures, mean and median scores were significantly lower at this point compared to baseline (data not shown). Floor and/or ceiling effects were observed for 5 scales (PF, RP, BP, VT and RE), but were not observed for the summary measures PCS and MCS. Scores for all scales and summary measures differed across KPS median. Table 4 presents MCID, SEM and SDC values using last available data. For PCS and MCS, SDCs and estimated MCIDs were similar to those obtained using baseline data.

**Table 4:** MCID, SEM and SDC estimations using the last available data (*N* = 73)

SF-36 scale	MCID	SEM	SDC	Responsiveness to change^a^	Floor/ceiling effect
PCS	6.3	1.52	4.21	Yes	None
MCS	8.7	2.54	7.03	Yes	None
PF	7.6	1.94	5.38	Yes	Floor effect
RP	6.1	1.71	4.75	Yes	Floor effect
BP	7.6	2.10	5.83	Yes	Ceiling effect
GH	7.5	3.52	9.75	No	None
VT	9.0	3.00	8.32	Yes	Floor effect
SF	7.1	4.04	11.20	No	None
RE	6.0	2.26	6.26	No	Ceiling effect
MH	11.3	3.85	10.66	Yes	None

*MCID* minimal clinically important difference, *SEM* standard error of measurement, *SDC* smallest detectable change

^a^Responsiveness to change was present if SDC < MCID

## Discussion

This study provides a robust assessment of responsiveness to change of SF-36, a widely used HRQoL assessment tool, in a population of patients with stage 5 CKD. It shows that whilst the usefulness of most SF-36 scales in assessing HRQoL in this population, especially over time, is largely limited by floor and/or ceiling effects or by poor responsiveness to change, the summary measures PCS and MCS are sensitive to change and free from floor or ceiling effects. For PCS, any change over time which is greater than 5.7 points is likely to be clinically significant. For MCS, this change must exceed 9.2 points.

SF-36 was originally designed as a HRQoL assessment tool for populations with chronic uncomplicated medical conditions [43, 55]. A particular concern was therefore that in this population of patients with an advanced life-limiting disease, substantial floor and/or ceiling effects would be found. This was not demonstrated. In fact, floor and/or ceiling effects at baseline were found for 3 scales: RP, RE and BP, similar to previous reports in different populations, including the one in which the tool was originally developed [55]. As the disease progressed though (measured here by the last available data), floor effects were also observed for the PF and VT scales, suggesting that in those with a short life expectancy, SF-36 scales may not be able to differentiate between the HRQoL of different individuals. The summary measures PCS and MCS did not, however, present a floor or ceiling effect in this population at any time. They may therefore be a more appropriate outcome measure in this population, yet must be considered in the context of the scales from which they are derived.

A second concern regarding the appropriateness of SF-36 in this population was its ability to detect clinically significant change over time. Previous studies using SF-36 in patients with advanced CKD reported changes smaller than 4 PCS points as statistically significant, without commenting on its clinical significance [6, 7]. We used several methods to specifically estimate the amount of change which would be clinically significant to patients (MCID), and compared it to the inherent accuracy of the tool (SDC) in this population. Terwee et al. [32] argue that for a tool to demonstrate acceptable responsiveness to change, SDC must be smaller than MCID. Applying this criterion, five of the eight SF-36 scales were found to be not responsive to change in this population, and a sixth had a ceiling effect at baseline. Therefore, only the summary measures (PCS and MCS) and their key contributors (PF and MH, respectively) provided acceptable data.

PCS and MCS were originally constructed in an attempt to aggregate SF-36 scales that have similar factor content, to simplify statistical testing and interpretation [44, 56]. Empirical data demonstrated their theoretical advantage in increasing the number of levels defined, decreasing confidence intervals, and eliminating floor and ceiling effects [56]. Those findings were replicated in our population of patients with advanced CKD, as demonstrated by freedom from floor and/or ceiling effects and good responsiveness to change for the summary measures. Importantly, results for PF and MH, the key contributors to PCS and MCS, respectively, mirror those of the summary measures, implying that those 2 domains, whether measured by a specific scale or by a summary measure, can be reliably measured in this population over time. Unfortunately, other domains of HRQoL (i.e. RP, BP, GH, VT, SF and RE) were not as reliably measureable in this population by this tool, as will be further discussed below.

Several methods were used to estimate the MCID. Of those, the anchor-based method of comparison to functional status produced the most stringent criteria, which also appeared to best fit previous results: unlike other potential anchors which were studied, baseline KPS scores significantly correlated with baseline PCS (data not shown). Previous studies of this population showed that KPS deteriorated as death approached [57], as might reasonably be expected for physical HRQoL. Estimating MCID using normative data appeared to be less useful. Since the same normative population was used for norming SF-36 scores and for this analysis, transformed (not normed) scores had to be used, and this limited comparability to SDC estimations (which were performed using normed scores) as well as to other populations. Also, MCID for the summary scores, including this study’s primary outcome PCS, could not be estimated.

Given the popularity of SF-36 as a HRQoL assessment tool in CKD and other populations, it is surprising that so little has been reported of its MCID. Key publications by the tool’s developers consistently avoided specifying any MCID [43, 44, 56, 58], as does the publicly accessible website (http://www.sf-36.org/). One user manual [59] was cited by others to indicate that ‘differences of 5.7 and 6.3 points for PCS and MCS scores, respectively, significant at the 95 % level, are considered clinically important’ [26]. We could not, however, obtain this manual to critically appraise the data on which this claim was based. Interestingly, for PCS this figure is identical to the estimation produced here.

Some authors have made assumptions regarding MCID: Luckett et al. [60] suggested that MCID should be 10 % of any given scale, but accepted that this was arbitrary. Others have chosen a 3–5 point difference as MCID for SF-36, with little justification [61, 62]. A review aimed at developing MCIDs concluded that for SF-36, MCID for all scales is 3–5 points, but this too was based on minimal data of questionable quality [63]. Those assumptions may be too lenient: Pagels et al. [3] showed that the mean difference in PCS scores between patients with CKD stages 2–3 to that of patients with CKD stages 4–5 was higher than 10 points, which suggests that a clinically meaningful change over time should at least match this figure. With a different HRQoL tool, a 5–10 % difference in scores was associated with ‘little’ patient-reported change over time, a 10–20 % difference with ‘moderate’ change and >20 % difference with ‘very much’ change [64]. Although this study was performed in a different population (cancer patients), and with a different tool (EORTC-QLQ-C30), its results do highlight that a patient felt change in HRQoL may be much larger than mean differences in scores which achieve statistical significance in a large sample.

Potential problems with focusing on MCIDs must be acknowledged. A widely used definition of MCID is ‘the smallest difference in score in the domain of interest which patients perceive as beneficial and which would mandate, in the absence of troublesome side effects and excessive cost, a change in the patient’s management’ [65]. Quantitative estimations of MCIDs, as described in our study, do not take into account cost-effectiveness. Furthermore, MCID can show significant between-population variation, even with the same tool [66]. Also, authors often fail to account for the impact of the chosen anchor (for anchor-based methods) or the sample (for distribution-based methods) on the estimated MCID [67]. Within-population variation was demonstrated in this study when different estimation methods were used and compared (Table 3). The magnitude of MCID may also vary according to baseline status (e.g. small improvements being more noticeable to those with poorer baseline HRQoL) and the direction of change (improvement vs. deterioration) [67, 68]. This was not accounted for in the current study. Finally, it should be acknowledged that it may be easier to demonstrate that a change of 3–5 points is clinically meaningful than to prove that a change of 1–2 points is not [67]. On another level, we note that MCID is a measure derived from populations, and as such, it averages scores and eliminates inter-individual variance. It may therefore not be suitable in assessing change in an individual over time.

A further limitation of this study is the choice of the reference population for producing normed SF-36 scores. As discussed, an ideal reference population was not available, and compromise had to be accepted. The WHS 2007 data were chosen on the basis of having been collected at a similar time to this study’s data collection, from a British general population. However, an alternative reference population could have been derived from the Health Survey for England 1996 [69, 70], which represented English people at an earlier time. A brief examination of this alternative reference population yielded overall lower scores on all SF-36 scales and summary measures (data not shown), suggesting that the choice of reference population could have influenced the MCID estimations as well.

This study recruited a relatively small convenience sample of conservatively managed individuals, who represent a minority of stage 5 CKD patients (10–20 % in the three recruiting renal units) [35]. However, data completeness was very high, and statistically significant results were obtained despite the use of highly conservative statistical methods (i.e. nonparametric tests and Bonferroni adjustment to multiple comparisons). The use of ANCOVA, a generally robust method to violations of normality, only marginally changed the non-adjusted results (data not shown). SEM is an inherently parametric construct but in our data yielded a less stringent estimation of MCID and was thus deemed less appropriate. That some of our results are comparable to previously published findings with SF-36 [55, 59] lends further strength to their generalizability.

This study was not designed to determine whether SF-36 is a valid measure for HRQoL assessment in this population of conservatively managed patients with stage 5 CKD. One can presume that those patients may have similar concerns to those of others with stage 5 CKD or to other palliative care populations, although this was not specifically sought or demonstrated. In stage 5 CKD, patients identified sexual functioning, body image, sleep and freedom or control as areas of importance [71, 72]. In palliative and end of life care, existential concerns, comfort and peace of mind were highlighted as important [73–75]. All of those areas are not covered by SF-36. The content validity of SF-36 as a generic tool has previously been established, but it is recognized that it may miss content areas of relevance to particular populations [43]. In CKD, this led to the development of the KDQOL, a dialysis-specific QoL questionnaire with an SF-36 core [71]. SF-36 was not previously used in the context of palliative care, possibly because it does not address the unique concerns of patients with advanced disease [76].

In conclusion, the appropriateness of SF-36 as a HRQoL assessment tool in patients with stage 5 CKD is limited both by its floor and ceiling effects and by its poor responsiveness to change in this population. In addition, it does not include domains which become increasingly important as illness advances (such as existential concerns, comfort and peace of mind). Only the summary measures of SF-36, PCS and MCS, and their key contributors PF and MH, respectively, can be used to assess changes in HRQoL over time. The minimal amount of change which is likely to be clinically meaningful is 5.7 for PCS and 9.2 for MCS, which is much higher than that used in similar populations so far.

Despite widespread use of SF-36 in patients with CKD, a robust assessment of its responsiveness to change in this population has never (to our knowledge) been reported. This study adds such an assessment, and its results call into question the usefulness of this outcome measure in this population. We believe that this information will be valuable both in selecting HRQoL measures for future studies, and for interpreting the findings of previous published studies. Future research should focus on assessing the validity of SF-36 in this population and should include global measures most relevant to populations with advancing illness, such as the Integrated Palliative Care Outcome Scale [77, 78], alongside HRQoL measures. Such an approach will open the door to research which could determine the effectiveness of interventions (e.g. palliative care) on the HRQoL of patients with stage 5 CKD.
